# Moller's Norwegian Cod Liver Oil

**Published:** 1886-04

**Authors:** 


					﻿Whoojini Couch Cured,
PAGE’S
VAPORIZER.
A simple yet per
feet apparatus
for Vaporizing
Cresolene. Creso-
lene vaporized in
a closed room
cures Whooping
Cough in a few
days. It is also
effectual in re-
lieving Asthma.
Croup, Diphtheria
and Scarlet Fever.
Ask your drug-
gist for it. Price,
$1.60.
W. 11. SehieiTelin & Co.. Role Agents,
170 William St., New York.
				

